# Increased pulse wave velocity and augmentation index after isometric handgrip exercise in patients with coronary artery disease

**DOI:** 10.1186/s40885-015-0016-7

**Published:** 2015-04-23

**Authors:** Shin-Hang Moon, Jae-Cheol Moon, Da-Hee Heo, Young-Hyup Lim, Joon-Hyouk Choi, Song-Yi Kim, Ki-Seok Kim, Seung-Jae Joo

**Affiliations:** Cardiology Division, Department of Internal Medicine, Jeju National University Hospital, 15 Aran 13-gil, Jeju City, Jeju Province 690-767 Republic of Korea

**Keywords:** Arterial stiffness, Aortic pressure, Pulse wave velocity, Coronary artery disease, Isometric exercise

## Abstract

**Background:**

Arterial stiffness of patients with coronary artery disease (CAD), which is expected to be increased due to a generalized atherosclerotic process of human body, may be more evident after the acute increase of blood pressure (BP) or peripheral vascular resistance. Isometric handgrip exercise is a simple and easily applicable method to achieve this goal. We investigated the changes of hemodynamic parameters and arterial stiffness indexes after handgrip exercise in patients with CAD.

**Methods:**

Forty-two subjects, who underwent coronary angiography (CAG), were enrolled. After CAG, baseline arterial waveforms were traced at the aortic root and external iliac artery using right coronary catheters. Arterial waveforms were recorded at 1, 2, and 3 min in the aortic root and at 3 min in the external iliac artery after isometric handgrip exercise at 30% ~ 40% of the maximal handgrip power. Augmentation pressure (AP) and augmentation index (AIx) were measured at aortic pressure waveforms. Pulse wave velocity (PWV) was calculated using the ECG-gated time difference of the upstroke of arterial waveforms and the distance between the aortic root and the external iliac artery.

**Results:**

Thirty patients had CAD (CAD group), and others showed no significant coronary stenosis (non-CAD group). Baseline hemodynamic parameters including AIx and PWV were not different between both groups. After isometric handgrip exercise, central systolic blood pressure (BP), central diastolic BP, central pulse pressure, peripheral systolic BP, and peripheral pulse pressure were increased in all patients. AIx inclined significantly from 1 min after exercise only in patients with CAD (before 17.7% ± 9.7% vs. 3 min after exercise 22.3% ± 10.7%, *p* < 0.001). PWV also increased significantly after exercise only in patients with CAD (before 10.03 ± 1.99 m/s vs. 3 min after 11.09 ± 2.45 m/s, *p* < 0.001).

**Conclusions:**

Arterial stiffness indexes at rest were not different between patients with and without CAD. After isometric handgrip exercise, increased arterial stiffness became evident only in patients with CAD.

## Introduction

Vascular aging is characterized by the functional and structural changes of the arterial wall, resulting in the increased arterial stiffness. Increased arterial stiffness is associated with the increased systolic blood pressure (BP) but decreased diastolic BP, which causes the widened pulse pressure and the decreased coronary perfusion. Arterial stiffness is known to be a useful marker for predicting the future cardiovascular events, such as myocardial infarction or stroke [1-4].

Arterial stiffness can be invasively or non-invasively evaluated. One commonly used method is measuring pulse wave velocity (PWV) between the aortic root or carotid artery and femoral artery, and increased PWV means stiff arteries [5]. A number of studies showed the increased aortic PWV in patients with coronary artery disease (CAD) [6-10]. Aortic PWV is regarded as a tool for detecting subclinical target organ damage in hypertensive patients [11].

Another method to assess the arterial stiffness is the analysis of pulse waveforms of the central arteries. The backward reflected wave from the peripheral arteries as well as the forward wave generated by the left ventricular contraction contributes to the central aortic pressure. As the arterial stiffness increases, the velocities of both forward and backward arterial waves increase, resulting in the earlier arrival of the reflected wave and the augmentation of the aortic systolic BP. This augmentation is expressed as augmentation pressure (AP) or augmentation index (AIx) which is a percentage of AP on pulse pressure [5]. The earlier studies showed that AIx was increased in patients with CAD [7,12]; but in recent studies, AIx was not associated with CAD, especially in elderly patients [9,13,14]. A subtle change of AP or AIx in patients with CAD may become more evident after the acute increase of systolic BP or peripheral vascular resistance by influencing the velocities of forward and backward waves.

Isometric handgrip exercise is an easily applicable maneuver to increase the cardiac afterload [15,16]. The usual response to isometric handgrip exercise is the increase in systolic BP and heart rate. The change of central aortic pressure waveform after isometric handgrip exercise may be variable according to the atherosclerotic status, but it has been rarely investigated in patients with CAD. We evaluated the changes of hemodynamic parameters and arterial stiffness indexes after isometric handgrip exercise in patients with CAD.

## Methods

### Study patients

Patients who underwent the coronary angiography (CAG) for the diagnosis of CAD or follow up after percutaneous coronary intervention (PCI) and agreed to participate in the study were included. CAD was defined as ≥70% stenosis in at least one major epicardial coronary artery or a history of PCI or coronary artery bypass graft. Patients with acute coronary syndrome, left main or three-vessel CAD, or valvular heart disease were excluded. The study protocol was approved by the institutional review board of Jeju National University Hospital. We enrolled 42 patients: 30 patients with CAD (CAD group) and 12 patients without CAD (non-CAD group), and they all signed the informed consents.

### Study protocol

Before CAG, the maximal voluntary forearm contraction power was measured with a JAMAR dynamometer (Sammons Preston Rolyan, Nottinghamshire, UK), and a submaximal target at 30% ~ 40% of maximal handgrip power was used for 3-min isometric handgrip exercise.

**Figure 1:**
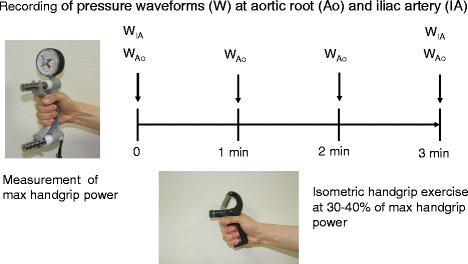
Study protocol. Baseline arterial pressure waveforms were traced at the aortic root (W_Ao_) and external iliac artery (W_IA_). A submaximal target at 30% ~ 40% of maximal handgrip power was used for isometric handgrip exercise. Arterial waveforms were recorded at 1, 2, and 3 min in the aortic root and at 3 min in the external iliac artery after exercise.

**Figure 2:**
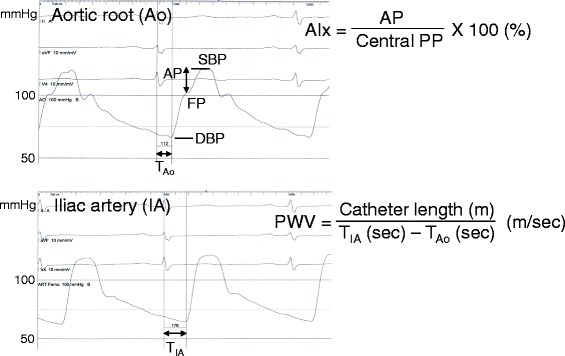
Measurements of augmentation pressure (AP), augmentation index (AIx), and pulse wave velocity (PWV). AP and AIx were measured at the central aortic waveforms. AP was maximal systolic blood pressure (SBP) minus forward pressure (FP). AIx was defined as AP divided by pulse pressure (PP) expressed as a percentage. PWV was calculated using the ECG-gated time difference of the upstroke of the arterial waveforms between the aortic root (T_Ao_) and the external iliac artery (T_IA_) and the distance between them measured using a catheter.

After routine CAG, baseline arterial pressure waveforms were traced at the aortic root and external iliac artery using a right coronary catheter and a fluid-filled pressure transducer system, and central and peripheral BP were measured from those waveforms. Arterial waveforms were recorded at 1, 2, and 3 min in the aortic root and at 3 min in the external iliac artery after isometric handgrip exercise (Figure 1). AP and AIx were measured at the central aortic waveforms. A merging point of the forward and the reflected waves was identified on recorded pressure waveforms. Forward pressure was defined as a pressure at the inflection point. AP was maximal systolic BP minus forward pressure. AIx was defined as AP divided by pulse pressure expressed as a percentage (Figure 2). In four patients with CAD, definite inflection points were not identified, and they were excluded from AP and AIx analysis. PWV was calculated using the ECG-gated time difference of the upstroke of the arterial waveforms and the distance between the aortic root and the external iliac artery which was determined by a tape measure of the catheter length from the tip to the entry point at an arterial sheath minus the length of an arterial sheath (12 cm) after removal of a coronary catheter (Figure 2).

### Statistics

Values are expressed as mean ±1 SD. Mean values between two groups were compared by Student’s *t*-test. Categorical variables were compared using chi-square test. Changes of hemodynamic parameters were evaluated by paired *t*-test, or where appropriate, Wilcoxon signed rank test. A *p* value <0.05 was considered statistically significant. All statistical analyses were performed using SPSS for window version 12 (SPSS Inc. Chicago, IL, US).

## Results

The mean age of patients of this study was 63.0 ± 9.3 years (range: 41–82 years). Age, sex, height, and body mass index were not different between patients with and without CAD (Table 1). Patients with smoking, hypertension, diabetes mellitus, and chronic kidney disease were not also different, but patients with hyperlipidemia were more common in CAD group. Laboratory findings including hemoglobin, fasting blood glucose, serum creatinine, serum LDL cholesterol, and hsCRP were not different, except higher HbA1c and lower total serum cholesterol in CAD group. Left ventricular hypertrophy by ECG criteria was similarly found in both groups. Patients with CAD were taking more aspirin and clopidogrel, but other medications including calcium channel blocker, angiotensin-converting enzyme inhibitor, angiotensin receptor blocker, beta-blocker, diuretics, alpha-blocker, nitrate, or statins were similarly prescribed.

**Table 1:** **Baseline characteristics of patients**

	**With CAD (** ***n*** **= 30)**	**Without CAD (** ***n*** **= 12)**	***p*** **value**
Age	63.4 ± 8.8	62.0 ± 10.8	0.656
Male	20 (66.7%)	8 (66.7%)	1.000
Height (cm)	161.8 ± 9.5	161.7 ± 8.2	0.958
Weight (kg)	65.8 ± 11.2	68.4 ± 14.0	0.523
Body mass index (kg/m^2^)	25.01 ± 2.90	25.99 ± 4.06	0.387
Smoker	10 (33.3%)	1 (8.3%)	0.247
Hypertension	23 (76.7%)	7 (58.3%)	0.274
Diabetes mellitus	9 (30.0%)	2 (16.7%)	0.464
Hyperlipidemia	28 (93.3%)	8 (66.7%)	0.046
Chronic kidney disease	3 (10.0%)	1 (8.3%)	1.000
Laboratory findings			
Hemoglobin (g/dL)	14.07 ± 1.78	13.58 ± 2.00	0.445
Fasting blood glucose (mg/dL)	109.9 ± 27.7	99.0 ± 14.80	0.206
HbA1c (%)	6.41 ± 0.82	5.97 ± 0.31	0.016
Creatinine (mg/dL)	1.22 ± 0.22	1.21 ± 0.32	0.924
Total cholesterol (mg/dL)	152.4 ± 29.7	175.4 ± 21.9	0.020
Triglyceride (mg/dL)	115.3 ± 52.5	128.5 ± 107.7	0.595
HDL cholesterol (mg/dL)	46.5 ± 10.7	50.4 ± 10.4	0.288
LDL cholesterol (mg/dL)	88.4 ± 24.2	102.4 ± 20.3	0.084
hsCRP (mg/dL)	0.240 ± 0.405	0.101 ± 0.101	0.270
LVH by ECG criteria	5 (16.7%)	2 (16.7%)	1.000
Medications			
Aspirin	21 (95.5%)	13 (68.4%)	0.036
Clopidogrel	17 (77.3%)	7 (36.8%)	0.012
Calcium channel blockers	12 (54.5%)	6 (31.6%)	0.209
ACEI	5 (22.7%)	4 (21.1%)	1.000
Angiotensin receptor blockers	6 (27.3%)	6 (31.6%)	1.000
Beta-blockers	13 (59.1%)	11 (57.9%)	1.000
Diuretics	4 (18.2%)	3 (15.8%)	1.000
Alpha-blockers	2 (9.1%)	3 (15.8%)	0.649
Nitrate	7 (31.8)	4 (21.1%)	0.499
Statins	20 (90.9%)	14 (73.7%)	0.219

*ACEI* angiotension-converting enzyme inhibitor, *CAD* coronary artery disease, *LVH* left ventricular hypertrophy.

Baseline central systolic and diastolic BP, pulse pressure, and forward pressure were not different between two groups (Table 2). AP and AIx of patients with CAD (9.3 ± 5.8 mmHg and 17.7% ± 9.7%) were not different from those of patients without CAD (10.0 ± 8.1 mmHg and 16.5% ± 12.2%). Peripheral systolic and diastolic BP and pulse pressure were not different. PWV of both groups was also not different between patients with CAD (10.03 ± 1.99 m/s) and without CAD (9.82 ± 1.10 m/s).

**Table 2:** **Baseline central and peripheral hemodynamic parameters and handgrip power**

	**With CAD (** ***n*** **= 30)**	**Without CAD (** ***n*** **= 12)**	***p*** **value**
Central hemodynamics			
Central HR (/min)	64.6 ± 9.1	65.1 ± 10.8	0.724
Central SBP (mmHg)	114.7 ± 18.1	124.6 ± 23.6	0.201
Central DBP (mmHg)	61.0 ± 7.7	68.1 ± 13.0	0.053
Central PP (mmHg)	53.7 ± 16.8	56.5 ± 19.1	0.702
FP (mmHg)	105.3 ± 17.3	114.6 ± 22.8	0.173
AP (mmHg)	9.3 ± 5.8	10.0 ± 8.1	0.765
AIx (%)	17.7 ± 9.7	16.5 ± 12.2	0.757
Peripheral hemodynamics			
Peripheral HR (/min)	65.1 ± 9.7	67.7 ± 12.5	0.451
Peripheral SBP (mmHg)	121.9 ± 18.8	131.8 ± 22.5	0.156
Peripheral DBP (mmHg)	60.2 ± 8.3	66.2 ± 12.5	0.079
Peripheral PP (mmHg)	61.7 ± 17.8	65.6 ± 19.2	0.536
PWV (m/s)	10.03 ± 1.99	9.82 ± 1.10	0.675
Isometric handgrip exercise			
Handgrip power, maximal (kg)	34.5 ± 12.7	36.6 ± 16.5	0.661
Handgrip power at exercise (kg)	12.7 ± 2.7	12.8 ± 2.8	0.944
Handgrip power %	38.7 ± 5.6	38.2 ± 9.1	0.821

*AIx* augmentation index, *AP* augmentation pressure, *CAD* coronary artery disease, *DBP* diastolic blood pressure, *FP* forward pressure, *HR* heart rate, *PP* pulse pressure, *PWV* pulse wave velocity, *SBP* systolic blood pressure.

**Figure 3:**
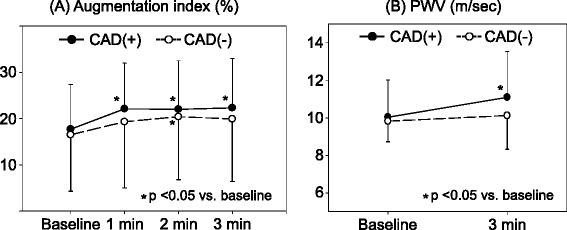
Changes of **(A)** augmentation index (AIx) and **(B)** pulse wave velocity (PWV) after isometric handgrip exercise in patients with or without coronary artery disease (CAD). AIx and PWV increased at 3 min after exercise only in patients with CAD. Values are mean ± SD.

Maximal handgrip power was not different. Handgrip power at exercise was about 38% of maximal power in both groups and also not different. During isometric handgrip exercise, heart rate, central systolic and diastolic BP, pulse pressure, and forward pressure were increased progressively from 1 min after exercise and reached the maximal level at 2 min in both groups (Table 3). AP and AIx of patients with CAD inclined significantly from 1 min after exercise (Figure 3). At 3 min, increases of AP (before 9.3 ± 5.8 mmHg vs. after 14.7 ± 8.3 mmHg, *p* < 0.001) and AIx (before 17.7% ± 9.7% vs. after 22.3% ± 10.7%, *p* < 0.001) were statistically significant. In patients without CAD, AP increased from 1 min after exercise, but AIx did not increase significantly at 1 and 3 min after exercise. At 3 min, AP (before 10.0 ± 8.1 mmHg vs. after 13.0 ± 9.3 mmHg, *p* = 0.084) and AIx (before 16.5% ± 12.2% vs. after 19.9% ± 13.5%, *p* = 0.172) of patients without CAD had not changed significantly.

**Table 3:** **Changes of hemodynamic parameters after isometric handgrip exercise**

	**Baseline**	**1 min**	**2 min**	**3 min**
Patients with CAD (*n* = 30)				
Central HR (/min)	64.6 ± 9.1	67.6 ± 9.9*	68.6 ± 9.8*	70.7 ± 13.3*
Central SBP (mmHg)	114.7 ± 18.1	132.0 ± 19.8*	136.1 ± 20.5*	137.0 ± 21.3*
Central DBP (mmHg)	61.0 ± 7.7	68.4 ± 8.4*	69.5 ± 8.4*	70.1 ± 9.1*
Central PP (mmHg)	53.7 ± 16.8	63.5 ± 17.6*	66.6 ± 19.0*	66.3 ± 20.4*
FP (mmHg)	105.3 ± 17.3	118.4 ± 18.1*	121.9 ± 19.0*	123.5 ± 19.8*
AP (mmHg)	9.3 ± 5.8	13.8 ± 7.0*	14.3 ± 7.7*	14.7 ± 8.3*
AIx (%)	17.7 ± 9.7	22.1 ± 9.9*	22.0 ± 10.5*	22.3 ± 10.7*
Peripheral SBP (mmHg)	121.9 ± 18.8			141.5 ± 24.6*
Peripheral DBP (mmHg)	60.2 ± 8.3			64.0 ± 9.3*
Peripheral PP (mmHg)	61.7 ± 17.8			77.5 ± 4.0*
PWV (m/s)	10.03 ± 1.99			11.09 ± 2.45*
Patients without CAD (*n* = 12)				
Central HR (/min)	65.1 ± 10.8	69.0 ± 11.3*	70.9 ± 11.4*	72.0 ± 12.6*
Central SBP (mmHg)	124.6 ± 23.6	138.7 ± 25.0*	141.9 ± 27.2*	142.6 ± 27.1*
Central DBP (mmHg)	68.1 ± 13.0	75.0 ± 13.7*	76.3 ± 15.4*	76.8 ± 14.5*
Central PP (mmHg)	56.5 ± 19.1	63.7 ± 19.9*	65.7 ± 19.9*	65.8 ± 18.8*
FP (mmHg)	114.6 ± 22.8	125.8 ± 26.1*	128.2 ± 27.9*	129.6 ± 28.6*
AP (mmHg)	10.0 ± 8.1	12.9 ± 10.8*	13.8 ± 10.3*	13.0 ± 9.3
AIx (%)	16.5 ± 12.2	19.3 ± 14.3	20.4 ± 13.6*	19.9 ± 13.5
Peripheral SBP (mmHg)	131.8 ± 22.5			141.2 ± 29.1*
Peripheral DBP (mmHg)	66.2 ± 12.5			67.3 ± 13.1
Peripheral PP (mmHg)	65.6 ± 19.2			73.9 ± 22.2*
PWV (m/s)	9.82 ± 1.10			10.12 ± 1.79

*p < 0.05 vs. baseline.

*AIx* augmentation index, *AP* augmentation pressure, *CAD* coronary artery disease, *DBP* diastolic blood pressure, *FP* forward pressure, *HR* heart rate, *PP* pulse pressure, *PWV* pulse wave velocity, *SBP* systolic blood pressure.

Peripheral systolic BP and pulse pressure increased after 3-min isometric handgrip exercise in both groups (Table 3). PWV of patients with CAD increased significantly (before 10.03 ± 1.99 m/s vs. after 11.09 ± 2.45 m/s, *p* < 0.001), but PWV of patients without CAD had not changed after isometric handgrip exercise (before 9.82 ± 1.10 m/s vs. after 10.12 ± 1.79 m/s, *p* = 0.584) (Figure 3). No ischemic complications occurred during isometric handgrip exercise.

## Discussion

The major findings of this study are the following: (1) isometric handgrip exercise is a simple, easily applicable, and safe maneuver to increase the central aortic BP and pulse pressure; (2) PWV and AIx measured at rest may not be useful markers for CAD presence, especially in old aged group; and (3) the acute increase of systolic BP after isometric handgrip exercise may uncover a subtle change of PWV and AIx in patients with CAD.

Atherosclerosis is a generalized process of the arterial system, resulting in intimal hyperplasia, plaque formation, narrowing of the internal diameter, obstruction, or decreased compliance. Atherosclerotic change of large arteries such as aorta makes them stiffer. One of the most commonly used methods to evaluate the arterial stiffness is measuring aortic PWV. Aortic PWV is regarded a useful marker for the prediction of future CAD or stroke. One community-based study showed that subjects with the third tertile of aortic PWV had increased risk for CAD with hazard ratio (HR) 2.45 and stroke with HR 2.28 [1]. In the Framingham heart study, subjects with higher aortic PWV carried a 48% increase in the risk of cardiovascular disease such as myocardial infarction, unstable angina, heart failure, or stroke [2]. In meta-analysis, aortic PWV remained a predictor of CAD (HR: 1.23) and stroke (HR: 1.28) even after adjusting for conventional risk factors [4], and an increase of aortic PWV by 1 m/s corresponded to risk increase of 14%, 15%, and 15% in total cardiovascular (CV) events, CV mortality, and all-cause mortality, respectively [3]. Measuring PWV may be more useful when it predicts the presence and severity of CAD in the high risk group. Numerous studies showed that not only increased aortic PWV but also brachial-ankle PWV were associated with CAD [6-10,17,18], and aortic PWV was a major determinant of myocardial ischemic threshold in patients with moderate CAD [19].

AIx is another useful marker for increased arterial stiffness. In CAD, AIx was also increased, especially in younger patients [7,12], and it was associated with myocardial ischemic threshold [19]. However, AIx was not a predictor of future CV events in the Framingham heart study [2].

In this study, no differences were found in age, height, smoking, prevalence of hypertension, diabetes mellitus or chronic kidney disease, and medications which may influence PWV and AIx. BP of hypertensive patients was adequately controlled. However, all central and peripheral hemodynamic parameters at rest, including PWV and AIx, were not different between patients with and without CAD (Table 2). Arterial stiffness is heavily dependent on the aging process [5], and, therefore, increased arterial stiffness may become obscure in elderly patients with CAD. The mean age of this study was 63.4 ± 8.8 years in patients with CAD and 62.0 ± 10.8 years in patients without CAD. PWV and AIx measured at rest in this old aged group may not be useful indicators for CAD presence. The previous study showed that the power of aortic PWV predicting the future CV events decreased with age (HR: 1.89, 1.77, 1.36, and 1.23 for age ≤50, 51 to 60, 61 to 70, and >70 years, respectively; *P*_interaction_ <0.001) [4]. AIx was also a useful marker for the presence of CAD in younger patients with age ≤60 years but not in older patients with age >60 years [9,12], and it was related to the extent of coronary atherosclerosis only in patients with age ≤60 years [12]. Recent studies showed that AIx did not reflect the presence of CAD in elderly subjects [14,20].

Isometric handgrip exercise is a simple, easily applicable, and safe stress test. During sustained isometric handgrip exercise, heart rate, systolic and diastolic BP, left ventricular systolic and end-diastolic pressure, and cardiac output were increased [16,17,21]. In patients with CAD, it also increased heart rate, BP, total peripheral resistance, and cardiac output [22]. In a few studies, central hemodynamic parameters such as central systolic and diastolic BP, central pulse pressure or AIx, and PWV were all increased during isometric handgrip exercise in healthy subjects [23,24]. In this study, isometric handgrip exercise increased the heart rate, central systolic and diastolic BP, central pulse pressure, and forward pressure, and 2-min exercise was enough to reach plateau levels. However, a significant increase in AIx or PWV after a 3-min exercise was only found in patients with CAD. These findings suggest that a subtle difference of arterial stiffness in CAD may be uncovered after the acute increase of systolic BP, and isometric handgrip exercise is an effective maneuver to accomplish this goal.

This study has several limitations. First, instead of a catheter with a high-fidelity micromanometer, a fluid-filled catheter was used to record the pressure waveforms. Accurate pressure recording may be blunted in a fluid-filled transducer system. Definite inflection points were not identified in aortic pressure waveforms of four patients (9.5%) in this study, and they were excluded from pulse wave analysis. Second, most of the patients were taking medications with a vasodilating property. These medications might be a cause of no difference in AIx or PWV at rest between patients with and without CAD. It might also influence the response of PWV or AIx during isometric handgrip exercise, but such medications were similarly taken in both groups. Third, poor patient cooperation during isometric handgrip exercise may result in insufficient hemodynamic stress, but all patients had finished 3-min exercise at 30%–40% of maximal handgrip power. Fourth, the number of patients was too small, especially in non-CAD group, and this may result in the similar baseline AIx and PWV. Although statistically not significant, baseline central and peripheral BP was lower in patients with CAD. Because BP is one of the most important determinants of AIx and PWV, this finding may be another cause of no difference in baseline AIx and PWV between two groups.

All hemodynamic parameters were invasively measured after CAG. Feasibility of this invasive method in the usual clinical practice is very limited. A future study of measuring central hemodynamic parameters with an applanation tonometry during isometric handgrip exercise may solve this problem.

## Conclusion

Isometric handgrip exercise is a simple, easily applicable, and safe maneuver to increase the central aortic BP and pulse pressure. Arterial stiffness index such as PWV or AIx at rest was not different between patients with and without CAD, suggesting that PWV and AIx measured at rest may not be useful markers for CAD presence, especially in old age group. After isometric handgrip exercise, increased arterial stiffness became evident only in patients with CAD.

## Abbreviations

ACEI: angiotensin-converting enzyme inhibitor
AIx: augmentation index
AP: augmentation pressure
CAD: coronary artery disease
CAG: coronary angiography
CV: cardiovascular
DBP: diastolic blood pressure
FP: forward pressure
HR: hazard ratio
PCI: percutaneous coronary intervention
PP: pulse pressure
PWV: pulse wave velocity
SBP: systolic blood pressure
SD: standard deviation
